# Adapting the Wheelchair Skills Program for pediatric rehabilitation: recommendations from key stakeholders

**DOI:** 10.1186/s12887-021-02564-9

**Published:** 2021-03-01

**Authors:** Geneviève Daoust, Paula W. Rushton, Marissa Racine, Karolann Leduc, Najoua Assila, Louise Demers

**Affiliations:** 1 School of Rehabilitation, Faculty of Medicine, Université de Montréal, Montréal, Canada; 2 CHU Sainte-Justine Research Center, 5200 Bélanger Street, Montréal, Québec H1T 1C9 Canada; 3 School of Kinesiology and Physical Activity Sciences, Université de Montréal, Montréal, Canada; 4 Centre de Recherche de l’Institut Universitaire de Gériatrie de Montréal, Montréal, Canada

**Keywords:** Occupational therapy, Wheelchair skills program, Pediatric rehabilitation, Knowledge-to-action, Consolidated framework for implementation research, qualitative studies

## Abstract

**Background:**

Backed by over 20 years of research development, the Wheelchair Skills Program (WSP) has proven to be a safe and effective program to improving wheelchair skills for adult wheelchair users. However, evidence is lacking for the pediatric population, which may help to explain the limited use of the WSP in pediatric settings. While additional evidence specific to the pediatric population is needed, concurrent implementation of the WSP into pediatric clinical practice is equally prudent to allow those users to benefit from the years of accumulated WSP evidence. To facilitate implementation of evidence-based programs into practice, adaptation is also often required to improve the fit between the program and the local context. Therefore, the objective of this study was to understand what adaptations, if any, are required for the WSP to be implementable in a pediatric setting.

**Methods:**

A deductive qualitative descriptive study design was used, guided by the Knowledge to Action Framework and Consolidated Framework for Implementation Research (CFIR). Occupational Therapists (OTs) from a pediatric rehabilitation center and two specialized schools in Montreal, Canada were invited to participate in a 90-min focus group. The Framework Method was followed for the data analysis.

**Results:**

One focus group in each site (*n* = 3) was conducted with a total of 19 participants. From the OTs’ perspectives, our analysis revealed benefits of WSP use and various issues (e.g. some skills seem unrealistic) affecting its uptake in relation to the constructs of the CFIR Intervention Characteristics domain. The results provided guidance for the recommendations of adaptations (e.g. addition of a caregiver assistance score) to enhance implementation of the WSP in pediatric rehabilitation settings and helped to identify the need for the production of new knowledge and knowledge translation (KT) tools.

**Conclusions:**

Implementation of the WSP with the adaptations and KT tools proposed could allow pediatric manual wheelchair users to improve their wheelchair skills.

**Supplementary Information:**

The online version contains supplementary material available at 10.1186/s12887-021-02564-9.

## Background

Independent mobility among pediatric manual wheelchair users is important for their achievement of developmental milestones [1, 2], yet many children using a manual wheelchair rely on their parents and others for personal mobility [3, 4]. As with most technologies, simply providing a manual wheelchair does not guarantee its safe and effective use.

One way to improve mobility is through wheelchair skills training using an evidence-based program, such as the Wheelchair Skills Program (WSP) [5]. The WSP includes assessment tools (i.e., the Wheelchair Skills Test [WST] and the Wheelchair Skills Test Questionnaire [WST-Q]) and a training guide (i.e., the Wheelchair Skills Training Program [WSTP] that can be used to test and train a set of over 30 manual wheelchair skills progressing from indoor to community and advanced levels. Backed by over 20 years of research development, there is extensive evidence that the WSP is a safe, effective intervention with the adult population [6]. Nevertheless, evidence is lacking for the pediatric population, which may help to explain the limited use of the WSP in pediatric settings [7, 8].

**Fig. 1:**
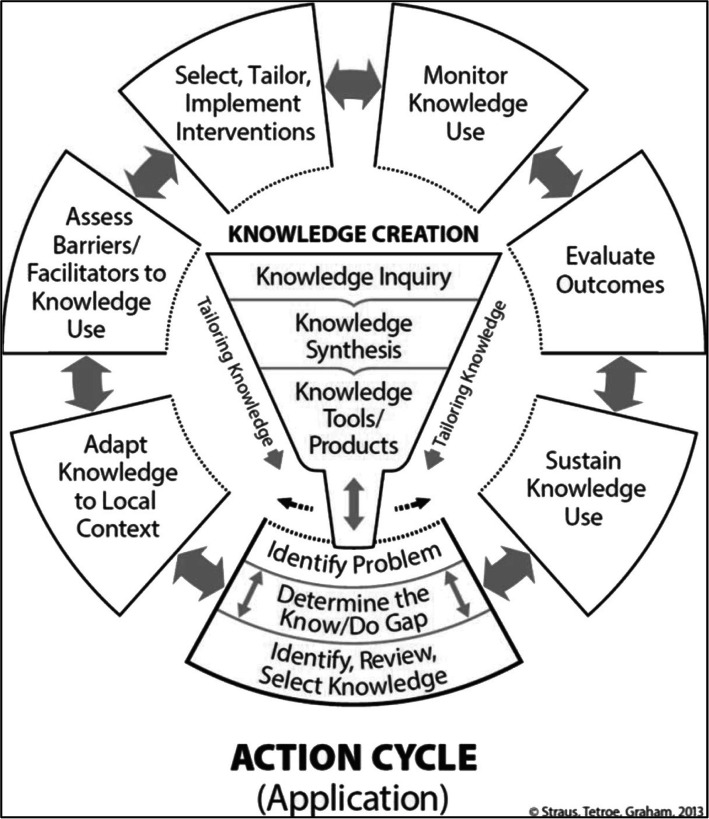
Knowledge to Action framework. Introduction Knowledge translation: What it is and what it isn’t. Knowledge Translation in Health Care 2013. p. 1–13. Reproduced with permission of John Wiley and Sons

As depicted in the Knowledge to Action (KTA) framework [9], the process for transferring research evidence (e.g., WSP evidence) into clinical practice (e.g., pediatric rehabilitation) involves both creating and applying knowledge (Fig. 1). The knowledge creation funnel of the KTA framework describes how a more refined, and likely more useful to the end users, generation of knowledge is produced as the knowledge passes through each stage (i.e. 1-knowledge inquiry, 2-synthesis, 3-tools and/or products). Considering the knowledge creation stages of the KTA framework from the perspective of the WSP with the pediatric population could bring insights of the knowledge gaps for this population.

The first generation WSP knowledge (i.e. knowledge inquiry) provides strong evidence for its use with adult and older adult populations but is limited for the pediatric population. The effectiveness of wheelchair skills training using the WSP has been demonstrated in 52 publications including 16 randomized controlled trials (RCTs) (https://wheelchairskillsprogram.ca/en/publications-impact/). However, only one study [10] specifically targeted children. Similarly, there have been 85 PubMed-referenced papers (https://wheelchairskillsprogram.ca/en/publications-impact/) either about the Wheelchair Skills Test and Wheelchair Skills Test Questionnaire or that have used these measures as outcomes. However, there is only one by M Huegel, et al. [11] that was specific to pediatrics.

In terms of the second generation WSP knowledge (i.e. synthesis) there have been two systematic reviews and meta-analyses that provide empirically sound evidence for the application of the WSP in clinical practice with adults and older adults [6, 12]. However, there were no published RCTs with pediatric wheelchair users that could be included in these systematic reviews.

Finally, the third generation WSP knowledge (i.e. products and tools), important for facilitating clinical uptake, is correspondingly focused on adults and older adults with few references to pediatrics in the WSP Manual [5] and the absence of knowledge products (e.g. poster, videos) targeting pediatric manual wheelchair users (PMWUs) or their parents on the WSP website (wheelchairskillsprogram.com).

From the knowledge-creation perspective, gaps in WSP use with the pediatric population highlights the need to circle back to the production of first, second and third generation WSP knowledge creation. Indeed, encouraging researchers to produce additional evidence specific to PMWUs is important for effective application of the WSP in this population. However, concurrent implementation of the WSP into pediatric clinical practice is equally prudent to allow PMWUs to benefit from the years of accumulated WSP evidence. This notion is particularly important given the likelihood that the new evidence will result in the addition of pediatric-specific considerations, a fine-tuning for this population, while the core WSP components will remain the same. Representative of the fluid nature of the boundaries between the knowledge creation and the action cycle, we suggest that an exploration into the adaptation (action cycle step 2) of the WSP is warranted in order to inform further knowledge creation and to facilitate its implementation into pediatric clinical practice. Thus, the objective of this study was to understand what adaptations, if any, are required for the WSP to be implementable in a pediatric setting.

## Methods

### Design

A deductive qualitative descriptive study design [13] was conducted using focus groups. This study was approved by the Sainte-Justine University Hospital Research Center Ethics Board and all participants provided written informed consent. Participant salaries were reimbursed to the organizations to compensate for study participation. The Consolidated criteria for reporting qualitative research (COREQ) checklist was used to facilitate comprehensive reporting of this study [14].

### Guiding conceptual frameworks

In addition to the KTA framework, the Consolidated Framework for Implementation Research (CFIR) [15] was used to provide insight into factors that may influence implementation outcomes [16]. The CFIR provides a listing of 39 constructs organized into five domains (i.e., intervention characteristics, inner setting, outer setting, characteristics of individuals involved and process) which have been associated with effective implementation. To answer our study objective, only the constructs referring to the *Intervention Characteristics* domain were used. Those constructs are: 1-intervention source (i.e. perception of key stakeholders about whether the intervention is externally or internally developed), 2-evidence strength and quality (i.e. stakeholders’ perceptions of the quality and validity of evidence supporting the belief that the intervention will have desired outcomes); 3-relative advantage (i.e. stakeholders’ perception of the advantage of implementing the intervention versus an alternative solution.); 4-adaptability (i.e. the degree to which an intervention can be adapted, tailored, refined, or reinvented to meet local needs); 5-trialability [i.e. the ability to test the intervention on a small scale in the organization, and to be able to reverse course (undo implementation) if warranted]; 6-complexity (i.e. perceived difficulty of the intervention, reflected by duration, scope, radicalness, disruptiveness, centrality, and intricacy and number of steps required to implement); 7-design quality & packaging (i.e. Perceived excellence in how the intervention is bundled, presented, and assembled); and 8-cost (i.e. costs of the intervention and costs associated with implementing the intervention including investment, supply, and opportunity costs) [15]. Detailed description of each construct can be found on the CFIR website (https://cfirguide.org). Those constructs guided the data collection by informing the development of the semi-structured interview guide questions and probes, and analysis by informing the deductive approach to data analysis.

### Research team

All team members were bilingual (English and French) and trained in qualitative research methods. The data were collected by three members of the research team (GD, MR and KL) and analyzed by all team members. Many of the study participants had existing work relationships with one team member (GD) who had previously been an occupational therapist at two of the three study sites. Study participants were aware that one member of the research team (PWR) was a co-developer of the program under study (i.e., the Wheelchair Skills Program).

### Settings

The study was conducted in one pediatric rehabilitation center, one specialized elementary school, and one specialized high school in Montreal, Canada. These settings provide services in the areas of rehabilitation, integration and social participation for children and teenagers with physical disabilities (≤ 18 years old at the rehabilitation center and ≤ 21 years old at the high school). Through their affiliation with a Mother-Child university hospital and being dedicated to pediatric wheelchair users, these settings are considered to provide more specialized pediatric rehabilitation services than what could be offered in most other centers in Quebec. They are also primary sites where approximately 650 children seek wheelchair-related services (i.e., new provision, adjustment and repairs) annually. Clinicians responsible for wheelchair provision and training in those settings are primarily OTs, thus significant WSP end-users. The primary spoken language is French in all settings.

### Participants and recruitment

At the time of the study, 40, 9 and 6 OTs were employed at the rehabilitation center, elementary and high school respectively. Potential participants were a sample of convenience. OTs were recruited through a short study presentation during an OT staff meeting at each site, followed by a Letter of Information sent via email distribution lists. OTs were eligible to participate if they had at least 2 months of experience at one of the study sites and were currently or had previously provided intervention to PMWUs.

### Procedure

A 90-min focus group was conducted at each site (*n* = 3) with the aim of determining what adaptations, if any, to the WSP would be useful to enhance its implementation in pediatric-rehabilitation settings. All focus groups were conducted in the French language by a moderator (GD) and assistant moderator (MR or KL), using the following process: welcome and introduction of moderator and assistant, including a description of their respective roles, discussion of the ‘ground rules’ for the focus group, a 30-min PowerPoint presentation regarding the WSP, which included a video of a full Wheelchair Skills Test (WST) administration of an adult wheelchair user from the WSP website, a facilitated discussion using a semi-structured focus group guide and a summary and wrap up.

The semi-structured focus group guide was developed by the research team based on the CFIR constructs from the Intervention Characteristics domain. After pilot testing the guide, it ultimately consisted of 10 open-ended questions, each with a set of potential probes (Supplementary file 1). Samples questions included: *‘How do you perceive the items in the Wheelchair Skills Test in terms of their use with the pediatric population? Are there items that you would adapt for your clients?* (adaptability), *‘What do you think about the format of the Wheelchair Skills Test? Is there another type of format or resources that may facilitate its use with the pediatric population?’* (design quality and packaging).

Each focus group was audio recorded and transcribed verbatim. All participants completed a sociodemographic questionnaire (e.g., age, years of experience) and rated their level of familiarity regarding the WSP from the following choices: ‘none, I have never heard of the program’, ‘a little, I know the big picture of this program’, ‘moderate, I know the program but I’m not an expert’, ‘excellent, I am very familiar with this program.’ Here, familiarity with the WSP was not specifically referring to the level of experience in using the program.

### Data analysis

The Framework Method [17] was used to deductively analyze the focus group data. This method was appropriate for this study as the technique is not aligned with any specific epistemological stance, rather it places the research question at the forefront of the analysis. Our analysis consisted of five steps. First, the three focus groups were transcribed verbatim by three members of the team (GD, MR and KL). Second, each team member familiarized herself with each focus group and recorded any initial thoughts and impressions. Third, a working analytical framework was developed with the 8 constructs of the *Intervention Characteristics* domain of the CFIR framework in order to identify in the data the WSP characteristic affecting its uptake in pediatric rehabilitation. Application of the analytical scheme was conducted using QSR International’s NVivo 12 software (www.qsrinternational.com) to index the transcripts according to the construct descriptions. At least 2 members of the research team applied the framework to each transcript. Fourth, the data were charted into a condensed matrix, which included both summarized data and pertinent illustrative quotes. This step was also conducted by at least 2 members of the research team. The summarized matrices provided a visual representation of the data in order to establish the emerging themes. As a fifth and final step, the data were interpreted with the summarized matrixes to respond to our initial research aim. Five team meetings with a total duration of ~ 25 h were required for the qualitative data analysis. Neither the focus groups transcripts nor the analyses were returned to the participants for pragmatic reasons (e.g., study timeline and participant burden) and data saturation was not sought.

## Results

Participants (*n* = 19) were 7 OTs from the rehabilitation center, 7 OTs from the elementary school and 5 OTs from the high school (18 females, 1 male). Participant demographics are presented in Table 1 and all names are pseudonyms. Participants on average had 12 years of experience working with PMWUs, but with a range from 0.3 to 31.2 years. Nine (47%) participants were ‘a little’ and 9 (47%) were ‘moderately’ familiar with the WSP, with only one (5%) OT in the elementary school group reporting to be ‘very familiar’ with the program.

**Table 1:** Participant demographics (*n* = 19)

Pseudonym	Age range (years)	Gender	Highest education level	Experience in pediatric (years)	Experience with PMWUS (years)	Familiarity with the WSP
	Mean = 38,4 (SD = 11.6)			Mean = 14,3 (SD = 11.6)	Mean = 12,0 (SD = 12.2)	
Alice	NP	Female	Bachelor	12,7	12,7	Moderate
Sandra	30–39	Female	Master^a^	5,4	0,5	Moderate
Lena	20–29	Female	Master^a^	4,1	4,1	Some
Kayla	20–29	Female	Master^a^	1	0,3	Moderate
Gabrielle	30–39	Female	Bachelor	11,4	10	Some
Nicole	40–49	Female	Bachelor	16	0,6	Some
Jacqueline	50–59	Female	Bachelor	31,1	31,1	Some
Evelyne	40–49	Female	Bachelor	25,5	10	Moderate
Magalie	20–29	Female	Master^a^	1	0,5	Excellent
Marie-Eve	20–29	Female	Master^a^	2,6	1	Some
Noémie	40–49	Female	Bachelor	20,5	20,5	Moderate
Manon	50–59	Female	Bachelor	34	34	Moderate
Florence	20–29	Female	Master^a^	3	1	Some
Johanne	50–59	Female	Bachelor	29	29	Some
Angela	40–49	Female	Bachelor	17	17	Some
Suzie	20–29	Female	Master^a^	4,4	4,4	Moderate
Robert	50–59	Male	Bachelor	31,2	31,2	Moderate
Caroline	30–39	Female	Master^a^	7,4	7,4	Moderate

^a^Professional Master. *PMWUs* Pediatric manual wheelchair users, *NP* Not provided, *SD* Standard deviation. Level of familiarity from the response choice: ‘none, I have never heard of the program’, ‘a little, I know the big picture of this program’, ‘moderate, I know the program but I’m not an expert’, ‘excellent, I am very familiar with this program’

Our qualitative analysis revealed benefits of using the WSP and various issues affecting its uptake in pediatric settings in relation to five constructs of the CFIR Intervention Characteristic domain (i.e. relative advantage, adaptability, complexity, evidence strength & quality and design quality & packaging). There were no data that were categorized within the constructs of intervention source, trialability and cost. A summary of the WSP implementation issues presented throughout the results section with their associated CFIR constructs is shown in Table 2. Direct quotes provided in this article were translated from French to English by bilingual members of the research team.

**Table 2:** Proposed WSP adaptation for the pediatric rehabilitation context

WSP Implementation Issues	CFIR Intervention Characteristic Construct	Proposed adaptation	KTA framework Need for Knowledge Creation?	
			Knowledge Inquiry (1st generation knowledge)	KT Tool / Product (3rd generation knowledge)
WST needs to be adapted to the pediatric population (requires a more playful approach for young PMWCUs; developmental considerations)	Adaptability	Requires Knowledge Inquiry	WST measurement property study with PMWCUs and parents	Narrated WST PMWU/Parent videos
		Addition of ‘*special considerations for children*’ in WST scoring guide		N/A
Some skills seem unrealistic for PMWUs and more applicable for parents	Adaptability	Requires Knowledge Inquiry	Developmental acquisition of wheelchair skills	Case studies, Training videos with PMWUs
		Addition of the existing caregiver assistance score column in the WST form	WST measurement property study with the added caregiver assistance score	N/A
Difficulty to use the WSP for training children	Adaptability	Addition of ‘*special considerations for children*’ in WSP Manual	1) Intervention study with PMWUs and parents; 2) Suggestions from clinical experts	N/A
WST-Q too complex for self-administration with PMWCUs	Complexity & Design Quality and Packaging	Requires Knowledge Inquiry	WST-Q measurement property study with PMWUs	N/A
		Development of a tablet-based administration format		
WSP Manual is dense and detailed and thus difficult to quickly access pediatric-specific information	Complexity & Design Quality and Packaging	Optimization of tabs and internal links in the WSP Manual to facilitate easy access	N/A	Condensed pediatric-specific version of the WSP Manual
PMWU training of community and advanced skills frequently avoided due to safety concerns and perceptions that it’s not possible or not important	Evidence Strength and Quality	Addition of ‘*special considerations for children*’ in WSP training guide	Intervention study with PMWUs and parents	Pediatric-specific training posters, narrated training videos, training workbook, case studies and storybook
Unavailability (i.e., logistic and emotional) of parents to participate in assessment and training	Design Quality and Packaging	WST-Q in an electronic fillable format	N/A	Narrated parent-specific training videos, ‘train at home’ guide, training plan template, section on the WSP website or YouTube Channel
		Requires KT Tool / Product Development		
Perplexity on how to engage the parent for training	Design Quality and Packaging	N/A	N/A	Parent-specific promotional materials (e.g., pamphlet, promotional video)

*WSP* Wheelchair Skill Program. *CFIR* Consolidated Framework for Implementation Research. *PMWCU* Pediatric Manual Wheelchair User. *WSTP* Wheelchair Skill Training Program. *WST* Wheelchair Skills Test *WST-Q* Wheelchair Skills Test Questionnaire. *KT* Knowledge Translation

### Relative advantage

According to this construct, OTs perception of the advantage of implementing the WSP versus an alternative solution will be a positive aspect for future implementation. In this case most OTs agreed that the Wheelchair Skills Test (WST) would be valuable to implement in practice as they named many benefits of using this standardized tool compared to maintaining current practice (i.e., unstructured observation). By using the WST, Jacqueline found that she would *“be able to name more specific objectives”* and Evelyne said it would *“help in the wheelchair selection and provide more information when recommending the transition to power mobility.”* Magalie recognized that the WST “*can help to structure the assessment and make sure we don’t forget anything”* and Florence added that it would *“standardize the evaluation practice in the school.”* Further, some OTs agreed that use of the WST with the parents could be a facilitator to improve their participation for training because parents would be informed in the beginning of the possible challenges with wheelchair use.

*“By doing the wheelchair [skills] evaluation, you see what the obstacles are and you understand what the challenges are with the equipment ... therefore the parents would be more aware of what is coming [and] would be more aware of the possible challenges in the environment ... we may have a better participation afterward.” -Nicole.*

Similar benefits were also identified regarding the use of the WSP for training. OTs from all groups agreed that using the WSP could ensure a more thorough wheelchair skills training which could help both PMWUs and their parents to use the wheelchair more effectively and decrease the amount of time dedicated to wheelchair repairs.

### Adaptability

Adaptability refers to the degree to which the WSP can be adapted, tailored, refined, or reinvented to meet the needs of the pediatric population. Here, the OTs perceived that the WSP is not perfectly adapted or tailored to the pediatric population needs. First, OTs treating preschool children found the actual performance-based WST lacked playfulness and suggested that a more playful approach to the administration would help the younger PMWUs to understand the instructions and to engage in the process more easily. Sandra, who treats children with cerebral palsy, expressed that the performance-based test would be challenging by saying*, “[ … ]for them to understand the task of turning right and left around the pylons, for sure they (PMWUs) will just run into them, they don’t understand so easily and there’s the impulsiveness too.”* She followed by proposing a fun obstacle path to the test, *“it could have a path or some sort with a story like a princess who goes looking for something [ …*] *it might be fun and increase collaboration.”* She referred to the administration of the Assisting Hand-Assessment [18] for her proposed example since she did not, and neither had the other OTs in her group, experienced or adapted the WST with the younger PMWUs.

Secondly, some OTs expressed hesitation to use the WST because of the lack of detail about the developmental progression in the acquisition of skills or other developmental milestones (e.g. motor or cognitive skills) to accompany the scoring criteria. The qualitative observation during a skill seems also to be more relevant for the OTs as described by Jacqueline, “*… it is really how the child does it that is more important than the distance he or she can make.”*

Other OTs wondered about the applicability of testing certain skills, given the differences between children and adults in wheelchair size and configuration. For example, Kayla questioned the feasibility of PMWUs doing the *ascends high curb* skill given the size of the wheelchair and its components (e.g., does the wheelchair have a long enough wheelbase to allow successful completion of this skill?). Similarly, Noémie described that the skill *picks object from floor* is not achievable for most of her PMWUs at the elementary school because *“they never or rarely have a short floor to seat height that allows them to do that.”* Through that reflection, OTs in her group suggested that some skills in the WST could be removed to be more accessible to their clientele. Similar concerns were voiced regarding training that some skills were too unreachable for PMWUCs. In other cases, some OTs mentioned that caregiver training may be more applicable than training the PMWU him/herself, such as in case of neuromuscular disease.

*“There are a lot of items that are so confronting for a child with muscular weakness, like transfer to the ground, fold the wheelchair … so … I think that it could be really good to do the training with the caregiver … even just ascending a slight incline can be very difficult for some users.” - Laurie-Anne.*

### Complexity due to the design quality and packaging

Complexity refers to the perceived level of difficulty of the WSP or the perceived difficulty to implement the WSP. Design quality and packaging refers to the perceived excellence in how the WSP is bundled, presented, and assembled. In this case the WSP design quality and packaging affected the perception of complexity to use it. First, OTs found that the general scoring criteria description on the performance-based WST form makes the scoring ambiguous and adds an extra step into the scoring as they need to refer back to the information in the manual. Also, OTs commented on how the self-report Wheelchair Skills Test Questionnaire (WST-Q) as presented seems to be too complicated for the PMWUs to self-administer the test. Since many PMWUs have cognitive and perceptual problems, it would require supervision and guidance by the OT to complete the test and despite this, doubted the accuracy of the child’s responses. For those reasons, they perceived the questionnaire impractical.

*“Honestly it will be difficult for students to read the question, find their way in the form, follow each line and answer the 4 questions well. And to differentiate between what they can do vs. their confidence; it is difficult for them to make this nuance. I think it would be long and would require supervision.” - Caroline.*

Regarding training, some OTs expressed that the absence of concrete guidance for pediatric users in the training guide makes it harder to know how to adapt the intervention or to give the adequate tips for a PMWU even when they have the knowledge of the proper techniques.

*“There are more obvious skills like rolling forward that with our kids can be really long and arduous and so I did not feel prepared to train this skill. I know where the hands should be on the wheels, how to push, the cycles, but with a child I felt a little more resourceless when I was staying at the rolling forward skill for a long time.” - Magalie.*

Thus, OTs felt that the enhancement of the presentation of some WSP tools would increase their competencies to use the program and in the same way decrease the complexity.

Regarding the caregiver, although OTs expressed that caregiver training may be more relevant in some cases, they named several challenges to involve the parents which seemed also to be associated with the WSP design and presentation. The first barrier to caregiver training mentioned by the OTs concerned the parents’ limited availability. Specifically, even if parents are in favor of wheelchair skills training, their participation may be limited due to the other rehabilitation demands as Lena explained, *“… the percentage [of parents] that would accept [wheelchair skills training] would not even be 5% because they are so overwhelmed by all the appointments at the rehabilitation center …*” . The OTs in the school settings also explained that the absence of parents is simply the reality of school settings where parents are rarely involved during school hours.

*“There is a whole section in the WSP that is to teach the caregiver and I would say that this would be a challenge for us because we do not have the parents at school and most of the time when you prescribe a MWC you do not see the parents much ... we often give a lot of information over the phone...” -Suzie.*

The other barrier was expressed in the form of a discomfort to suggest wheelchair skills training such as described by Sandra, *“Maybe parents would accept manual wheelchair skills training, but since they know how to manipulate a stroller … , they may feel patronized when training is proposed.”* Other OTs noticed that parents could experience difficulty to first accept the manual wheelchair recommendation, resulting sometimes in a lack of involvement throughout the wheelchair provision process. In that regard, Alice reported that*, “They [parents] don’t want to hear about manual wheelchair skills training. They prefer to push their child [in the wheelchair] because they keep hoping they will walk again.”* Gabrielle added that *“[MWC skills training] is really not their priority, it’s more about safety or how to fold [the MWC] … or academic prerequisites [needed] in school …”* .

When discussing how the WSP tools (e.g. website, video, forms) could be used to reach out parents outside the rehabilitation settings, Sandra suggested that an electronic fillable format of the WST-Q could be more convenient over the actual printable form, since email communication with the parents is common. Still, OTs felt that the way videos are presented seemed more appropriate for clinical use and wouldn’t feel comfortable to send them to the parent without any specific guidance. OTs agreed that more dedicated tools to reach out the parents and to facilitate the approach could increase their involvement for wheelchair skills training.

### Evidence strength and quality

This construct discusses how OTs perceived the evidence supporting the belief that the WSP will have desired outcomes. This construct was especially relevant for the community and advanced skills in the WSP. Indeed, OTs expressed reservations for training the community and advanced skills with PMWUs because of the limited literature supporting the benefits of training those skills with PMWUs in conjunction with limited feedback from PMWUs and clinical experience. Although OTs considered the wheelie-related skills necessary in adulthood, many expressed safety concerns for PMWUs which prevented them from conducting training of these skills.

*“We want to make sure that there is no risk of falling or tipping over, so the anti-tippers are always kept in place. I have the impression that it is over time when the child matures, when he/she decided to remove the anti-tipper, well it would be correct, but for a elementary school age child, we will do everything to ensure safety.” - Marie-Eve.*

OTs in the elementary school group perceived that PMWUs should always have the anti-tippers to ensure their safety and training the wheelie should be done when the child is more mature. However, OTs in the high school group expressed similar uncertainties to conduct wheelie related skills training as they emphasized safety concern in relation to the adolescent judgement.

*“Maybe he is capable of doing a wheelie … but would I let him do it outside, since it is completely different. Would he be able to judge when to cross the street? Is it the right time? So even if he is physically able, often it is much more complex with our students.” - Suzie.*

Thus, the absence of WSP evidence specific to community and advanced skills with the pediatric population seems to have led to the perception that those skills just apply to adult MWUs and be too unsafe for PMWUs. In addition, the way in which the environment and adults compensate for a PMWU’s wheelchair skill deficits influenced OTs’ perception for the need to conduct thorough training. As mentioned by Manon, the community and advanced skills are not as important because the *“parents will push their child outside anyway.”*

Further rationale was conveyed by the OTs in the high school and rehabilitation center groups in terms of wanting to avoid confronting their clients with their mobility deficits and potential failure to perform a wheelchair skill. Instead, the OTs described relying on parents to compensate for a lack of skill by the parent performing the wheelchair skill, rather than the child. In school setting, Manon described examples of accommodating behaviors from the school staff towards *PMWU’s “… in the corridors, it’s incredible … all adults get out of the way to avoid being hit [by a PMWU], whereas the kids don’t even think that they should adjust their path.”* The staff habits to compensate for PMWUs may mask the need to train even the more basic skills. Therefore, the absence of WSP evidence specific to community and advanced skills with the pediatric population may lead to the perception that PMWUs will naturally depend on the adults to compensate their mobility limitation instead of learning how to be more independent.

## Discussion

When moving evidence-based programs into practice, adaptation is often required to improve the fit between the program and the local context (e.g., specific population needs, priorities, policies, resources) [19]. This study was the first to explore the perspectives of OTs regarding what adaptations, if any, to the WSP would be useful to enhance its implementation in pediatric-rehabilitation settings. Analyses of our qualitative data using the CFIR Intervention Characteristics domain provided guidance to identify recommendations of adaptations to respond to the study objective. Through the reflection, particular attention was given in order to find a balance between what can be adapted versus what should stay consistent to the actual WSP. Further, use of the Knowledge to Action (KTA) framework was useful in determining that many of the perceptions of the OTs required not adaptations to the program, but rather *3rd Generation WSP Knowledge* (KT tools and products), or *1st Generation WSP Knowledge* that will inform adaptations. A summary of the recommendations provided throughout the discussion are presented in Table 2.

To begin, OTs perceived that the WSP was not perfectly adapted and tailored to meet the pediatric population’s needs in terms of playfulness and developmental considerations. Although play is considered a significant occupation for children [20], the suggestion of a more playful approach to the administration of the Wheelchair Skills Test (WST) seems to contradict the prevalent use by pediatric OTs of norm-referenced and criterion-referenced standardized assessments [21, 22]. Indeed, play is predominantly used by OTs as a therapeutic tool [23], while the use of play-based assessments is infrequent [23, 24]. Further, studies that have used the WST with children [10, 11] have not reported the need to consider a more playful administration. While acknowledging the importance of play, but also considering the limited evidence to support the need to adapt the WST in a playful administration, we propose that a playful WST administration be considered as a need for new *first generation WSP knowledge* in terms of the measurement properties of the WST for the pediatric population, taking into account various pediatric age groups, diagnoses and developmental levels.

With regards to the need for developmental consideration in the WST, an important change in the scoring scale was made in Versions WST 5.0 and 5.1 of the WSP to increase the sensitivity and indirectly its applicability with PMWUs. Specifically, the 3-point response scale of previous versions [25] was changed to a 4-point scale (3 = advanced pass; 2 = pass; 1 = partial pass; 0 = fail) offering a more granular progression into each skill. For example, for the skill *gets over obstacle*, the child can now get a partial-pass score if he/she can get the casters over the obstacle but not the rear wheels, thus making the skill more accessible for younger or new wheelchair users. Similar to adults, various cognitive and physical abilities could influence how long it takes a PMWU to learn a skill and the level of skill acquired. Thus, norm-referencing the WST would not be relevant. However, in comparison with the adult population there may be certain aspects of normal child development that could influence how a child performs a skill which may need to be considered in the assessment. For example, do we have the same expectation in terms of propulsion pattern between a two-year old and an 8-year old wheelchair user? Is there an estimated age where we can expect a child to have the sufficient coordination to rise the front wheels to go over an obstacle? In Version 5.1 of the WSP Manual [26], a section called ‘*special considerations for pediatric wheelchair users’* is being added to both the testing and training sections for each skill which will be populated over time based on new evidence and clinical experience. These considerations for the WST adaptation also highlight the need for new *first generation WSP knowledge* in terms of the measurement properties for the pediatric population and exploration regarding the developmental progression of wheelchair skills acquisition, also questioned by M Huegel, et al. [11].

Regarding the applicability of certain skills in the WST, OTs suggested the removal of skills perceived to be inaccessible for the PMWUs. However, we think that this approach may limit perception of the need for training (PMWUs or their parents), and their potential progress. Perhaps a solution that would meet the needs of pediatric clinicians could be a modification in the presentation of the WST form. Explicitly, the already proposed *Caregiver assistance score,* which is now only presented in the WSP manual, could be added directly to the WST form. Addition of this 6-point score (5 = no assistance; 4 = stand-by assistance; 3 = verbal assistance; 2 = one-person physical assistance; 1 = two-person physical assistance; 0 = equipment needed) to the form itself may suggest more intuitively to include the parent in the test and provide assistance for skills that may be too hard for a child at a certain age. It may also provide needed quantification of assistance that can help to demonstrate progress over time which may not be reflected in the wheelchair skills score. Ultimately, collection of this information may facilitate an enhanced understanding of the pediatric continuum of wheelchair skills acquisition. The WSP Manual and Forms are already provided online in Word format and encourage customization to meet the needs of specific groups. Customization of the WST forms to also include more details in the scoring criteria could be a solution to answer the comments regarding the ambiguity with the general scoring guidelines and avoid the extra step of referring back to the Manual.

Modification in the presentation of the Wheelchair Skills Test Questionnaire (WST-Q) form to decrease its complexity and enable the self-administration could be done through the development of a tablet-based format. As children and adolescents are building insight on their capacities, self-administration of the WST-Q by PMWUs by may be interesting to promote self-determination over learned helplessness [27]. These considerations for WST-Q adaption also highlight the need for new *first generation WSP knowledge* in terms of the measurement properties for the pediatric population. Until then, modification of the WST-Q form into an electronic fillable format could be a simple solution to facilitate the use of the questionnaire with the parent answering as a “proxy”.

To address the OTs’ perspectives regarding the complexity to perform wheelchair skills training with PMWUs, addition of developmental considerations, pediatric-specific motor learning principles and training tips in the training guide could be potential solutions. For example, training tips for one-arm drive wheelchairs would be helpful as this type of propulsion is often recommended for children (as opposed to hand-foot propulsion technique). Sections have been added to Version 5.1 of the WSP Manual called *special considerations for pediatric wheelchair users*. Because the WSP manual is already dense and detailed, suggestion to optimize the presentation (e.g. table, tabs, internal link) or to create a condensed pediatric-specific version like the already developed condensed version for caregivers could facilitate the access of the pertinent information. The WSP Editorial Committee encourages such customization of the Manual’s content.

OTs preferred that PMWUs kept their anti-tippers and avoided to train community and advanced wheelchair skills because of safety concerns. It is true that children may be safer when they keep the anti-tipper but at the same time restricted when faced to certain environmental obstacles (e.g. curb). Training skills can permit the anti-tips to be removed and be more autonomous in different mobility situations. Provision of training for community and advanced wheelchair skills among PMWUs is also important given that such training, when transitioning into adult rehabilitation services, is not always available [7]. To promote WSP uptake for training community and advanced skills in pediatric rehabilitation, the identified concerns regarding safety issues cannot be dismissed. In fact, it determines the need for new *first generation WSP knowledge* in terms of effectiveness of the WSP in improving wheelchair skills among the pediatric population. It also emphasizes the need for *third generation WSP knowledge* in terms of the creation of pediatric-specific educational resources and knowledge translation materials that portray the use of the program with PMWUs (e.g., pediatric-specific case studies, narrated videos, training workbooks, posters).

Finally, our findings suggest that promotion of parent involvement is an important aspect of the WSP adaptability in order to facilitate the implementation in pediatric rehabilitation. One factor affecting involvement, however, is related to the uncertainty of OTs and the lack of tools to reach the parent for wheelchair skills training. As reported in the literature, clear and explicit information by service providers regarding interventions serves to positively influence parents’ expectations, thus facilitating engagement [28, 29]. We can hypothesize that the same would be required to engage parents in wheelchair skills training.

Given the demonstrated effectiveness of manual wheelchair skills training among caregivers [30, 31] and the associated benefits for the wheelchair user, parent involvement represents an important component of the wheelchair skills training process. This finding represents another avenue for the development of *third generation WSP knowledge*, with a caregiver focus (e.g., lesson plan wheelchair skills training templates for parents, a caregiver-specific section on the WSP website with targeted information such as ‘train at home’ guides, addition of caregiver-specific training videos). The availability of more targeted caregiver resources may facilitate a more informed approach by the OTs to encourage parent involvement, which may also serve to enhance parents’ acceptance of wheelchair skills training as a means of developing their child’s independence rather than a reinforcement of the losses related to the disability [29].

### Strengths and limitations

A strength of this study was the diversity in perspectives obtained from OTs working with PMWUs having different characteristics and ages, which was useful to see factors affecting WSP uptake into the whole pediatric age continuum. We believe that participation of these WSP end-users in the process of determining WSP adaptations can help to foster acceptance and ownership of the WSP in these pediatric settings [32]. Since the proposed recommendations do not necessarily address site-specific characteristics (e.g. organizational barriers), we are confident as to their generalizability to other pediatric settings.

Because suggested feedback to propose adaptations are more informative when participants have some experience with the program [33], a major limitation in this study was the fact that most OTs had not used the WSP clinically with PMWUs prior to the focus groups. Since OTs had not really used the WSP in practice, their feedback was based on impressions about the challenges to use the WSP with PMWUs, as opposed to ‘lived’ challenges. Despite this limitation, these impressions were valuable for the recommendations of 1st and *3rd Generation WSP Knowledge.* However, in terms of adaptations, many suggestions of adaptations lacked details. To obtain more detailed suggestions of adaptations, potential modification in the study method could have been to use the systematic adaptation method proposed by EK Chen, et al. [33] thus requiring each participating OT to use the assessment tools and conduct training with one PMWU in their caseload prior the study. Finally, we feel that participant reactions and suggestions may have targeted the testing component (i.e., WST and WST-Q), more than the training component (i.e., WSTP) of the WSP because at the beginning of the focus group a short orientation to the WSP presentation was provided which included a video of the WST administration. For future research, as the WSP implementation takes form, collecting additional feedback from OTs could help to provide answers to some of the remaining questions regarding the adaptations (i.e., playful approach to the WST, developmental progression). Considering that wheelchair skills training can include wide-ranging interventions that cannot be covered by a single discipline, solicitation of other rehabilitation professionals and school staff (e.g., physiotherapist, physical educator, social worker) may be useful in the WSP implementation.

## Conclusion

While highlighting the additional WSP knowledge needed with the pediatric population, this is the first study to identify potential adaptations that could promote WSP uptake in pediatric rehabilitation settings. Concurrent WSP research and implementation with the adaptations and knowledge translation tools proposed could be a next step to allow PMWUs to benefit from the years of WSP evidence. Thus, addressing this step is warranted for the implementation of the WSP into those pediatric settings.

## Supplementary Information

**Additional file 1 Supplementary file 1**. Focus group guide, Process and questions developed for the focus group.

## Data Availability

The data used in this study or a direct log-in in the NVivo database cannot be shared by the authors to respect participants’ confidentiality. The anonymous forms of datasets used and/or analysed during the current study are available from the corresponding author on reasonable request.

## Abbreviations

WSP: Wheelchair Skills Program
WST: Wheelchair Skills Test
WST-Q: Wheelchair Skills Test Questionnaire
WSTP: Wheelchair Skills Training Program
OT: Occupational therapist
PMWUs: Pediatric manual wheelchair users
KTA: Knowledge to action
CFIR: Consolidated framework for implementation research
KT: Knowledge translation
