# Supplementary material for: Adapting the Wheelchair Skills Program for pediatric rehabilitation: recommendations from key stakeholders
Source: BMC Pediatr. 2021 Mar 1;21:103. doi: 10.1186/s12887-021-02564-9 (PMC7919309; doi:10.1186/s12887-021-02564-9)
Supplement: Supplementary file 1 — Additional file 1 Supplementary file 1. Focus group guide, Process and questions developed for the focus group. [file 12887_2021_2564_MOESM1_ESM.docx]

**Focus group guide**

1. Welcome and introduction
2. PowerPoint Presentation
3. Description of the moderator and assistant moderator’s respective role including
4. Discussion of the ‘ground rules’ for the focus group
5. Questions for discussion:
6. I would like to start the discussion by going around the table to have everyone’s general impressions regarding the WSP. What do you think of the program from what you have experienced, know, or saw in the presentation?
7. How could the WSP better meet the needs in the delivery / follow-up of your clients compared to current interventions?
   1. Do you see any gaps that could be addressed by the WSP?
   2. What advantages would you see in using the WSP in general for [ex: your practice, the continuum of care, the implication of parents]
8. How do you find the items in the WST in terms of their use with the pediatric population?
   1. Are there items that you would adapt for your clients?
9. How do you fin the applicability of the program with the parents?
   1. Are there tools that you would adapt for them?
10. How do you find the level of complexity of the program in terms of its use with the pediatric population?
    1. Are there aspects of the program that would be more complex to use in your setting?
    2. Would there be the means to facilitate and / or reduce the level of complexity.
11. What do you think about the format of the [WST / WST-Q / WSTP]?
    1. Is there another type of format or resources that may facilitate its use with the pediatric population?
12. In general, can you tell us about how you choose the assessment tools, training programs and interventions?
    1. Usually, what influences your choice of assessment / intervention tools?
    2. What do you think of the scientific quality of the WSP?
    3. Does the scientific quality correspond to one of your selection criteria when choosing your assessment / intervention tools?
13. Acknowledging that the program was developed in another rehabilitation center and has been more used with the adult population, how does this influence your interest in the program?
14. How do you see the resources needed to use the program with respect to what is available in your setting?
    1. Are there things you would change in the program or you would need in your settings to facilitate its use?
15. Let’s say you wanted to implement the program in your setting, how would you begin?
    1. How do you see the idea to try the program in a small scale be applied in your context?

Abbreviations: WSP-Wheelchair skills Program. WST-Wheelchair Skills Test. WST-Q-Wheelchair Skills Test-Questionnaire. WSTP-Wheelchair Skills Training Program
